# Linking Uncultivated Microbial Populations and Benthic Carbon Turnover by Using Quantitative Stable Isotope Probing

**DOI:** 10.1128/AEM.01083-18

**Published:** 2018-08-31

**Authors:** Ömer K. Coskun, Monica Pichler, Sergio Vargas, Stuart Gilder, William D. Orsi

**Affiliations:** a Department of Earth and Environmental Sciences, Ludwig-Maximilians Universität München, Munich, Germany; b GeoBio-Center^LMU^, Ludwig-Maximilians-Universität München, Munich, Germany; Wageningen University

**Keywords:** microbial carbon cycling, DNA-SIP, qSIP, microbial dark matter, ammonia oxidation

## Abstract

Benthic environments harbor highly diverse and complex microbial communities that control carbon fluxes, but the role of specific uncultivated microbial groups in organic matter turnover is poorly understood. In this study, quantitative DNA stable isotope probing (DNA-qSIP) was used for the first time to link uncultivated populations of bacteria and archaea to carbon turnover in lacustrine surface sediments. After 1-week incubations in the dark with [^13^C]bicarbonate, DNA-qSIP showed that ammonia-oxidizing archaea (AOA) were the dominant active chemolithoautotrophs involved in the production of new organic matter. Natural ^13^C-labeled organic matter was then obtained by incubating sediments in the dark for 2.5 months with [^13^C]bicarbonate, followed by extraction and concentration of high-molecular-weight (HMW) (>50-kDa) organic matter. qSIP showed that the labeled organic matter was turned over within 1 week by 823 microbial populations (operational taxonomic units [OTUs]) affiliated primarily with heterotrophic Proteobacteria, Chloroflexi, Verrucomicrobia, and Bacteroidetes. However, several OTUs affiliated with the candidate microbial taxa Latescibacteria, Omnitrophica, Aminicentantes, Cloacimonates, *AC1*, Bathyarchaeota, and Woesearchaeota, groups known only from genomic signatures, also contributed to biomass turnover. Of these 823 labeled OTUs, 52% (primarily affiliated with Proteobacteria) also became labeled in 1-week incubations with [^13^C]bicarbonate, indicating that they turned over carbon faster than OTUs that were labeled only in incubations with ^13^C-labeled HMW organic matter. These taxa consisted primarily of uncultivated populations within the Firmicutes, Bacteroidetes, Verrucomicrobia, and Chloroflexi, highlighting their ecological importance. Our study helps define the role of several poorly understood, uncultivated microbial groups in the turnover of benthic carbon derived from “dark” primary production.

**IMPORTANCE** Little is known about the ecological role of uncultivated microbial populations in carbon turnover in benthic environments. To better understand this, we used quantitative stable isotope probing (qSIP) to quantify the abundance of diverse, specific groups of uncultivated bacteria and archaea involved in autotrophy and heterotrophy in a benthic lacustrine habitat. Our results provide quantitative evidence for active heterotrophic and autotrophic metabolism of several poorly understood microbial groups, thus demonstrating their relevance for carbon turnover in benthic settings. Archaeal ammonia oxidizers were significant drivers of *in situ* “dark” primary production supporting the growth of heterotrophic bacteria. These findings expand our understanding of the microbial populations within benthic food webs and the role of uncultivated microbes in benthic carbon turnover.

## Abstract (precis)

Little is known about the ecological role of uncultivated microbial populations in carbon turnover in benthic environments. To better understand this, we used quantitative stable isotope probing (qSIP) to quantify the abundance of diverse, specific groups of uncultivated bacteria and archaea involved in autotrophy and heterotrophy in a benthic lacustrine habitat. Our results provide quantitative evidence for active heterotrophic and autotrophic metabolism of several poorly understood microbial groups, thus demonstrating their relevance for carbon turnover in benthic settings. Archaeal ammonia oxidizers were significant drivers of *in situ* “dark” primary production supporting the growth of heterotrophic bacteria. These findings expand our understanding of the microbial populations within benthic food webs and the role of uncultivated microbes in benthic carbon turnover.

## INTRODUCTION

Freshwater ecosystems process large amounts of organic carbon, contributing significantly to global carbon fluxes as well as contributing 2.1 Pg C year^−1^ to greenhouse gas emissions (1, 2). A large data set from freshwater ecosystems indicates that freshwater emits at least 103 Tg CH_4_ year^−1^, which offsets 25% of the estimated land carbon sink (3). Although inland waters occupy a small fraction of the surface of the earth, these areas are active sites for the C cycle in terms of transportation, transformation, and storage (4). Microorganisms are likely the main players in the production and consumption of organic matter in these ecosystems (4). Microbial responses to organic matter are considered to be important in benthic habitats due to their quick reaction to fresh organic input (5) and their increased enzymatic activities, which serve as a starting point for the reworking and turnover of the deposited material (6).

The diversity and structure of bacterial communities in freshwater ecosystems indicate that Proteobacteria, Planctomycetes, Bacteroidetes, Chlorobi, Chloroflexi, Verrucomicrobia, and Nitrospira tend to be dominant (7). The specific composition of the communities depends largely on the environmental factors that govern the physiochemical character of the niche (8–10). The breakdown of terrestrially derived plant litter, dissolved organic matter from the catchment, wind-transported material, and algal growth are pivotal components of freshwater ecosystem functioning and thus serve as major organic matter sources (11). However, chemolithoautotrophic ammonia-oxidizing bacteria and archaea (AOB and AOA, respectively) that fix C *in situ* in sediments may also be an important C source for bacteria (12).

The full extent of microbial taxa driving benthic carbon turnover in benthic habitats remains poorly understood (13). Genome-centric studies have shown that uncultivated low-abundance microbes found in freshwater environments possess genes related to the degradation of amino acids and sugars, indicating a heterotrophic lifestyle for these groups (13). Although assembled genomes provide valuable insights into the metabolic capabilities and ecological roles of the many uncultivated microorganisms, no direct link between their metabolism and carbon turnover in freshwater sediments has been established.

In this study, we quantified the taxon-specific incorporation of ^13^C-labeled bicarbonate and the turnover of the resulting ^13^C-enriched organic matter so as to link uncultivated microbial populations to carbon turnover in freshwater sediments via quantitative stable isotope probing (qSIP) (14) We also tested the hypothesis put forward in genome-centric studies (13, 15) that low-abundance taxa play an active role in benthic carbon turnover. Our results demonstrated that carbon fixation was dominated by AOA, suggesting that they are a primary source of *in situ*-produced organic matter in the absence of light. We used the resulting ^13^C-enriched organic matter in qSIP experiments to quantitatively link the diversity of several low-abundance but ecologically important uncultivated groups, namely, Latescibacteria, Omnitrophica, Aminicentantes, Cloacimonates, *AC1*, Bathyarchaeota, and Woesearchaeota, to organic matter turnover in a benthic environment. Moreover, statistical tests from qSIP analyses allowed us to assess the distribution of microbial carbon turnover activities across bacterial and archaeal phylogenetic groups. Overall, these results showed that several uncultivated microbial taxa contribute actively to benthic carbon turnover derived from chemolithoautotrophic primary production in the absence of light.

## RESULTS

### ^13^C enrichment of organic matter.

**FIG 1:**
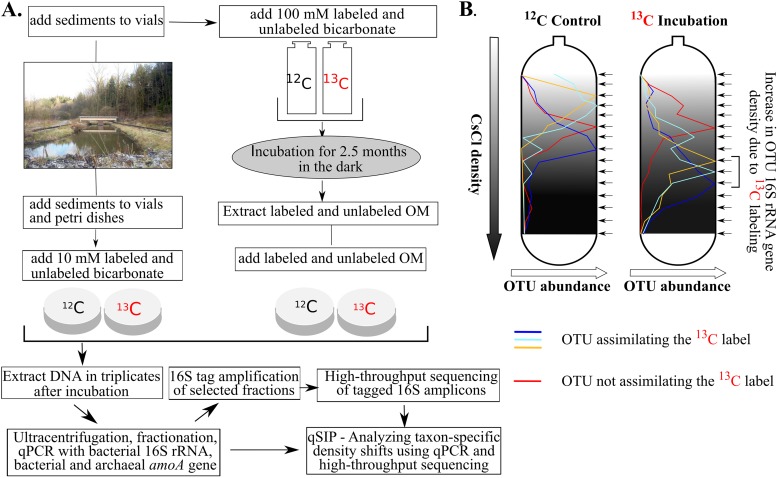
Flow diagram of the experimental setup. (A) Surface sediments were collected from a freshwater pond. Samples were amended with ^13^C-labeled sodium bicarbonate and were incubated in the dark in crimp-sealed glass vials for 2.5 months, and labeled organic matter (OM) was extracted. Afterwards, DNA-SIP microcosm studies were carried out in petri dishes using either the extracted HMW organic matter or bicarbonate. DNA was extracted in biological triplicate after 1 week and was subsequently used for qSIP. (B) Example of taxon-specific density shifts with OTUs assimilating or not assimilating the ^13^C label.

After a 2.5-month incubation with [^13^C]bicarbonate (Fig. 1), ^13^C enrichment in the DNA extracted from bulk sediment was clearly apparent in replicate incubations (see Fig. S1 in the supplemental material), with two separate peaks for the ^13^C-labeled incubation. The first peak, at ca. 1.70 g ml^−1^, had the same density as that for the unlabeled control incubation and thus likely came from unlabeled DNA. The second peak for the ^13^C-labeled incubation, at ca. 1.72 g ml^−1^, was not seen in the control incubation and thus likely came from labeled DNA of actively growing autotrophic microbes and the consumers of their biomass. In the organic matter extract that we added to the sediment (Fig. 1), the concentration of DNA was 3 μg g^−1^, and the atomic enrichment of ^13^C-labeled DNA was 30 to 40% (Fig. S1). Assuming that DNA accounts for ca. 3.1% of cell biomass (16), the total organic matter from cellular biomass added was ca. 99 μg g of sediment^−1^. Assuming that total biomass had a degree of labeling similar to that of DNA, we added ca. 35 μg of ^13^C-labeled biomass g^−1^ to our sediment samples for incubation. However, we note that these may be underestimates because biomolecules lower than 50 kDa were removed.

### Activities of ammonia-oxidizing archaea and bacteria.

**FIG 2:**
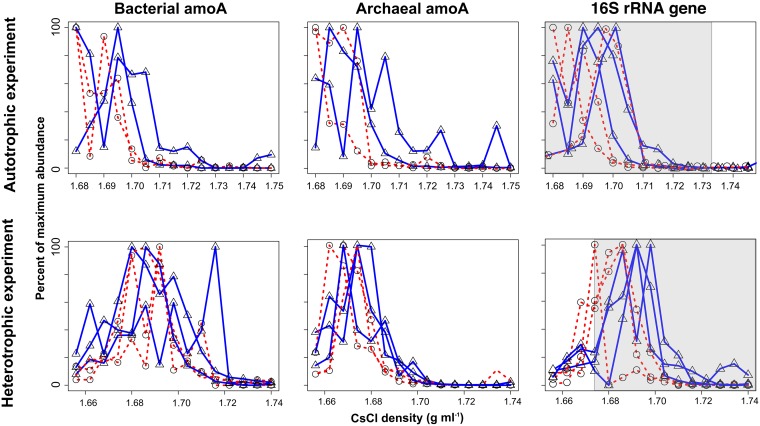
Quantification of archaeal and bacterial *amoA* genes and total 16S rRNA genes (using “universal” 16S rRNA primers) across CsCl density gradient fractions after the 1-week incubations. Solid blue lines with triangles represent ^13^C-labeled substrates, and dashed red lines with circles represent unlabeled substrates. The relative abundance of either 16S rRNA genes or *amoA* genes normalized to maximal abundance across all density fractions is shown along the *y* axis. Shaded areas represent the fractions sequenced for qSIP analysis.

Both bacterial and archaeal *amoA* genes exhibited isotopic labeling after 1 week in the presence of [^13^C]bicarbonate, with AOA exhibiting a shift of 0.006 g ml^−1^ and AOB exhibiting a shift of 0.006 to 0.012 g ml^−1^ (Fig. 2). Archaeal *amoA* genes (average, ∼10^6^ copies per g of sediment) were significantly more abundant than bacterial *amoA* genes (average, ∼0.3 × 10^5^ copies per g of sediment) in the sediments (see Fig. S2 in the supplemental material), indicating that they were the dominant chemolithoautotrophic ammonia oxidizers (17–20). This strongly indicated that AOA were actively growing and were a major source of carbon fixation and primary production in the sediments. With the exception of one replicate, there was no labeling of the bacterial *amoA* gene with ^13^C-enriched organic matter, whereas archaeal *amoA* genes showed a slight degree of labeling (Fig. 2). Operational taxonomic units (OTUs) found in the freshwater sediment were related to betaproteobacterial ammonia oxidizers observed in terrestrial environments, such as freshwater lakes, lake sediments, and soil (see Fig. S6 in the supplemental material).

### qSIP of bacterial and archaeal taxa.

Samples incubated for 1 week with ^13^C-labeled bicarbonate or HMW organic matter in air-tight, crimp-sealed glass vials showed minimal to no substrate uptake (see Fig. S3 in the supplemental material). However, in glass petri dishes sealed with gas-permeable paraffin, there was clear ^13^C labeling of 16S rRNA genes after 1 week (Fig. S3). The greater rate of DNA ^13^C labeling in the petri dishes was likely due to greater O_2_ diffusion into the sediments through the gas-permeable paraffin than with the gas-tight glass flasks that were crimp-sealed. Thus, we chose the density fractions for sequencing from samples incubated in petri dishes, since they more closely reflected the natural environment, where O_2_ diffuses into the overlying waters from the atmosphere, compared to incubations in gas-tight crimp-sealed vials.

The peaks in the buoyant density of 16S rRNA genes in the unlabeled control incubation ranged from 1.675 to 1.695 g ml^−1^ (Fig. 2; also Fig. S3 in the supplemental material), highlighting the biological variability between our replicate treatments. However, the range of these values is relatively low compared to those for incubations where ^13^C-labeled substrates were added: 1.691 to 1.705 g ml^−1^ for “autotrophic incubation” and 1.685 to 1.700 g ml^−1^ for “heterotrophic incubation” (Fig. 2). Thus, biological differences between replicates was not sufficient to mask reproducible ^13^C enrichment in our experiments.

**FIG 3:**
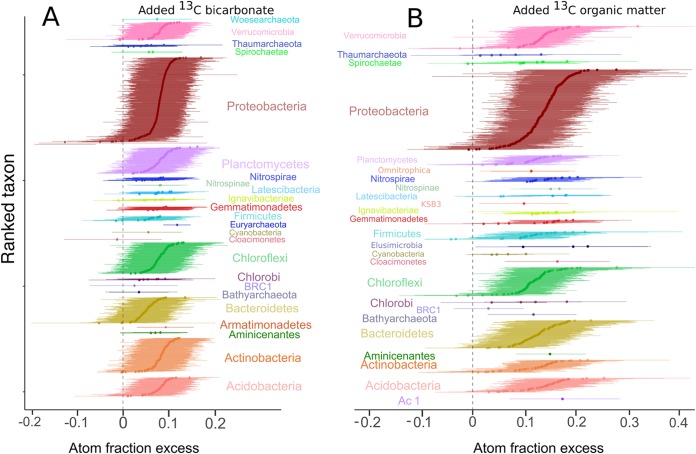
Taxon-specific shifts in the median atom fraction excess (^13^C) of OTUs with 90% confidence intervals. OTUs are color-coded by phylum. Excess atom fractions were caused by ^13^C assimilation from added ^13^C-labeled bicarbonate (A) or ^13^C-labeled organic matter (B). OTUs that do not overlap with zero are considered to be ^13^C labeled.

After incubation, the bacterial community from the bicarbonate incubation (1,236 OTUs) was dominated by the phyla Proteobacteria (58% of total sequences), Bacteroidetes (9%), and Verrucomicrobia (7%), while 931 OTUs from the HMW organic matter incubation were likewise dominated by several of the same phyla in nearly equal proportions (53% Proteobacteria, 9% Bacteroidetes, and 9% Chloroflexi) (see Fig. S4 in the supplemental material). A total of 798 OTUs (65% of the total) were labeled in the [^13^C]bicarbonate incubation, and 823 OTUs (88% of the total) were labeled in the ^13^C-labeled HMW organic matter incubation. Among these OTUs, the phyla Proteobacteria, Verrucomicrobia, Chloroflexi, and Bacteroidetes had the highest numbers of labeled OTUs in the ^13^C-labeled HMW organic matter incubation, whereas the phyla Proteobacteria, Acidobacteria, Actinobacteria, and Planctomycetes had the highest numbers of labeled OTUs in the [^13^C]bicarbonate incubation (Table 1; Fig. 3).

**TABLE 1:** Summary of taxa assimilating a ^13^C-labeled substrate after a 1-week period

Taxon	^13^C-enriched organic matter (heterotrophic assay)			^13^C-labeled bicarbonate (autotrophic assay)		
	Avg bootstrapped A median value^*a*^ of OTUs	No. of OTUs that did not overlap zero	No. of sequences (% of total)	Avg bootstrapped A median value of OTUs	No. of OTUs that did not overlap zero	No. of sequences (% of total)
Bacterial group						
*AC1*	0.174	1	191 (0.02)			
Acidobacteria	0.147	53	53,330 (4.29)	0.090	66	142,863 (8.36)
Actinobacteria	0.141	43	37,912 (3.05)	0.090	118	127,476 (7.46)
Aminicenantes	0.150	1	304 (0.02)	0.083	1	128 (0.01)
Armatimonadetes				0.095	1	193 (0.01)
Bacteroidetes	0.133	101	119,842 (9.6)	0.073	33	37,426 (2.19)
Chlorobi	0.132	3	1,063 (0.09)	0.076	2	459 (0.03)
Chloroflexi	0.125	108	122,862 (9.9)	0.090	90	116,303 (6.80)
Cloacimonetes	0.164	1	371 (0.03)			
Cyanobacteria	0.086	2	11,716 (0.94)			
Elusimicrobia	0.172	3	650 (0.05)			
Firmicutes	0.130	25	89,386 (7.19)	0.120	6	3,125 (0.18)
Gemmatimonadetes	0.157	13	7,543 (0.61)	0.073	11	16,020 (0.94)
Ignavibacteria	0.136	10	12,469 (1.00)	0.085	4	2,069 (0.12)
Modulibacteria (KSB3)	0.099	1	192 (0.02)			
Latescibacteria	0.170	5	2,202 (0.18)	0.089	10	6,282 (0.37)
Nitrospinae	0.160	2	6,309 (0.51)	0.083	2	5,335 (0.31)
Nitrospirae	0.146	18	17,753 (1.43)	0.082	11	22,886 (1.34)
Omnitrophica	0.113	2	1,922 (0.15)			
Planctomycetes	0.120	35	17,644 (1.42)	0.079	80	43,712 (2.56)
Proteobacteria	0.145	298	684,269 (55)	0.092	309	1,079,407 (63)
Spirochaetes	0.134	6	1,682 (0.14)			
Verrucomicrobia	0.138	89	51,909 (4.18)	0.087	52	105,543 (6.17)
Archaeal group						
Bathyarchaeota	0.118	1	862 (0.07)			
Euryarchaeota				0.087	1	307 (0.02)
Thaumarchaeota	0.108	2	526 (0.04)			
Woesearchaeota (DHVEG-6)				0.076	1	277 (0.02)
Total			1,242,909			1,709,811

a Median of bootstrapped excess atom fraction (EAF) values (see reference 14).

**FIG 4:**
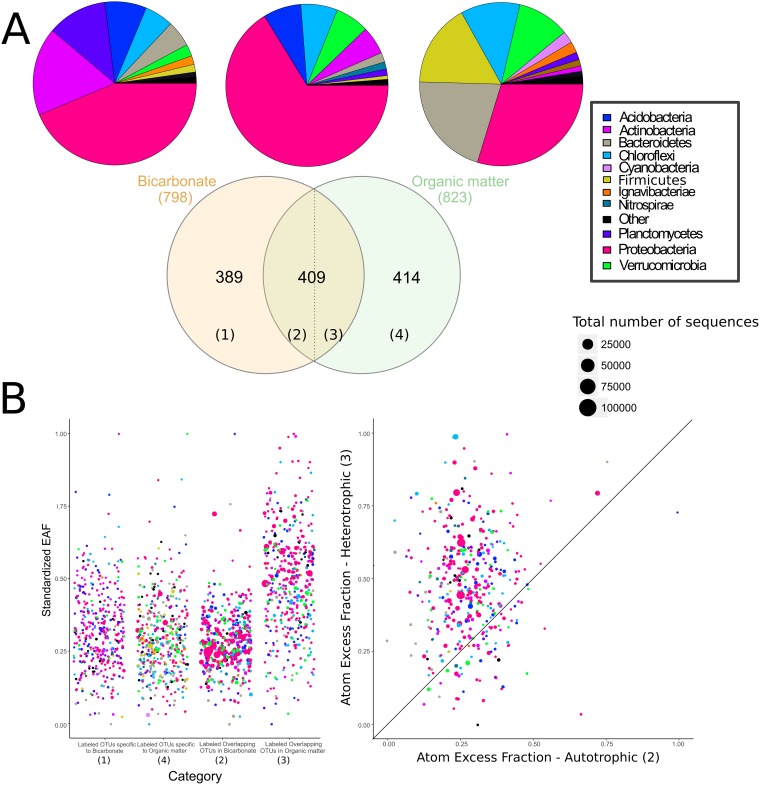
(A) Numbers of overlapping and nonoverlapping OTUs assimilating bicarbonate or organic matter, together with corresponding pie charts displaying the phylum-level composition of 16S rRNA gene sequences. Categories 2 and 3 in the overlapping region indicate different EAF values for OTUs labeled in the “heterotrophic” and “autotrophic” incubations. (B) Scatter plots of normalized EAF values (with 1 as the highest and 0 as the lowest value) for the labeled OTUs within four categories indicated in the Venn diagram in panel A. (Left) EAF values for all categories. (Right) *x-y* plot of category 2 versus category 3 (EAF values of overlapping OTUs in bicarbonate and HMW organic matter incubation, respectively.). The 1:1 line indicates OTUs that have the same rate of isotopic labeling in the bicarbonate and organic matter settings.

A total of 409 OTUs were labeled in both the [^13^C]bicarbonate and ^13^C-labeled HMW organic matter settings (Fig. 4A), represented predominantly by Proteobacteria (181 OTUs) and Acidobacteria (36 OTUs). In comparison, 389 OTUs were labeled exclusively in the incubations with bicarbonate, while 414 OTUs were labeled only in the ^13^C-labeled HMW organic matter incubation (Fig. 4A). Most of the OTUs detected in the bicarbonate incubations were related to known heterotrophic and or mixotrophic groups, whereas only 23 labeled OTUs were affiliated with known chemolithoautotrophic bacteria and archaeal nitrifiers.

## DISCUSSION

### Sources of chemolithoautotrophic production.

In agreement with studies from similar environments (see, e.g., reference 19), archaeal *amoA* genes were 15 to 19 times more abundant than bacterial *amoA* genes, suggesting that the former were numerically the most important primary producers in the absence of light (Fig. S2 in the supplemental material). Cell volumes of AOA are 10 to 100 times less than those of known AOB (21), and based on specific activity, AOB tend to dominate ammonia-oxidizing activity in soil if the AOA/AOB ratio is lower than 10 (22). Because the AOA/AOB ratio here is 15 to 19, our results suggest that AOA were as important a source of newly produced organic matter via carbon fixation in the dark as AOB, if not even more important. However, AOB had a higher level of atomic enrichment from ^13^C-labeled bicarbonate than AOA, indicating that AOB also played a role in carbon fixation in the dark (Fig. 3 and 4).

There was a small degree of AOA labeling in the ^13^C-labeled organic matter incubations (Fig. 2), which could be assigned to 9 OTUs affiliated with Thaumarchaeota (Fig. S6 in the supplemental material). Two of these OTUs were affiliated with clade I.b, known to exhibit mixotrophic activity (23), indicating that some Thaumarchaeota may have been performing mixotrophy in addition to strict autotrophy. Furthermore, some Thaumarchaeota in soil clade I.b are not obligate chemolithoautotrophs (24). It is therefore possible that our results show the activity of heterotrophic Thaumarchaeota.

### Estimating rates of carbon utilization.

The maximum amount of ^13^C labeling was twice as high in the ^13^C-labeled HMW organic matter incubation as in the [^13^C]bicarbonate incubation (excess atom fractions [EAF], ca. 0.4 versus 0.2 [Fig. 3]), indicating that microbial assimilation of carbon derived from HMW organic matter proceeded faster than that of carbon derived from dissolved inorganic carbon (DIC). The large fraction of labeled OTUs overlapping between heterotrophic and autotrophic incubations after 1 week (*n* = 409 [Fig. 4A]) suggests that these OTUs are capable of faster assimilation of DIC-derived carbon than the remaining 414 OTUs labeled in the ^13^C-labeled HMW organic matter incubation (Fig. 4A). These 409 overlapping OTUs thus are the microorganisms that either (i) consumed AOA and AOB necromass or biomass (and other chemolithoautotrophs) within 1 week after these autotrophs assimilated the [^13^C]bicarbonate or (ii) performed mixotrophy and also acquired some of the [^13^C]bicarbonate as a carbon source.

Rather than referring to these overlapping OTUs (*n* = 409) explicitly as mixotrophs or heterotrophs, we compared the EAF of all labeled OTUs (*n* = 823) by separating them into four categories: category 1, labeled OTUs specific to the bicarbonate incubation; category 2, “overlapping” labeled OTUs in the bicarbonate incubation; category 3, “overlapping” labeled OTUs in the HMW organic matter incubation; category 4, labeled OTUs specific to the HMW organic matter incubation (Fig. 4A). Using the EAF values as a proxy for the rate of carbon utilization (25), we were able to compare the taxa that were fastest and slowest at metabolizing the labeled substrate within each of these four categories. This showed that “overlapping” ^13^C-labeled OTUs (*n* = 409) had higher EAF values in the ^13^C-labeled HMW organic matter incubations (0.14 ± 0.03) than in the [^13^C]bicarbonate incubations (0.08 ± 0.01) (Fig. 4B). Thus, these faster-growing OTUs could assimilate carbon (EAF values, 0.051 to 0.165) from freshly produced “dark” primary production after 1 week yet utilized it much more (EAF values, 0.063 to 0.224) in a readily available HMW form (Fig. 4B).

The 409 overlapping OTUs had relatively high EAF values of >0.22 and were affiliated primarily with Alphaproteobacteria and uncultured Cytophaga-Flavobacterium genera within Alphaproteobacteria, Actinobacteria, and Gammaproteobacteria: namely, the Hyphomicrobium, Mycobacterium, and Amaricoccus genera and the order Xanthomonadales. The relatively greater enrichment of HMW organic matter by Alphaproteobacteria and Cytophaga-Flavobacterium is consistent with another study that showed that these groups utilize HMW chitin, *N*-acetylglucosamine, and protein (26). In soil, several taxa affiliated with Bacteroidetes have been shown to exhibit a copiotrophic lifestyle (27), a finding that is also consistent with our results.

Several of the overlapping OTUs (EAF, >0.12) were affiliated with uncultivated clades, including subgroups 11, 17, 18, and 22 in Acidobacteria, MB-A2-108 in Actinobacteria, VC2.1, Bac22, and VadinHA17 in Bacteroidetes, and SJA-15, SBR2076, and KD4-96 in Chloroflexi. This indicates a relatively greater enrichment of HMW organic carbon assimilation in these OTUs than in the “nonoverlapping” OTUs (Fig. 4A). Of those taxa, the Chloroflexi KD4-96 clade has been proposed to be involved in soils derived from fumaroles assimilating volcanic CO_2_ (28)—indicating its potential for ^13^C labeling by direct carbon fixation. The remaining 414 nonoverlapping OTUs in the HMW organic matter incubations consisted of numerous low-abundance undefined taxa with generally lower-than-average EAF values, such as the PLA 4 lineage, OM190, and vadinHA49 in Planctomycetes, BD2-2, WCHB1-32, and SB-5 in Bacteroidetes, and MSBL3 in Verrucomicrobia. Hence, these organisms had relatively lower rates of organic carbon uptake.

### Phylogenetic distribution of ^13^C-labeled taxa.

**FIG 5:**
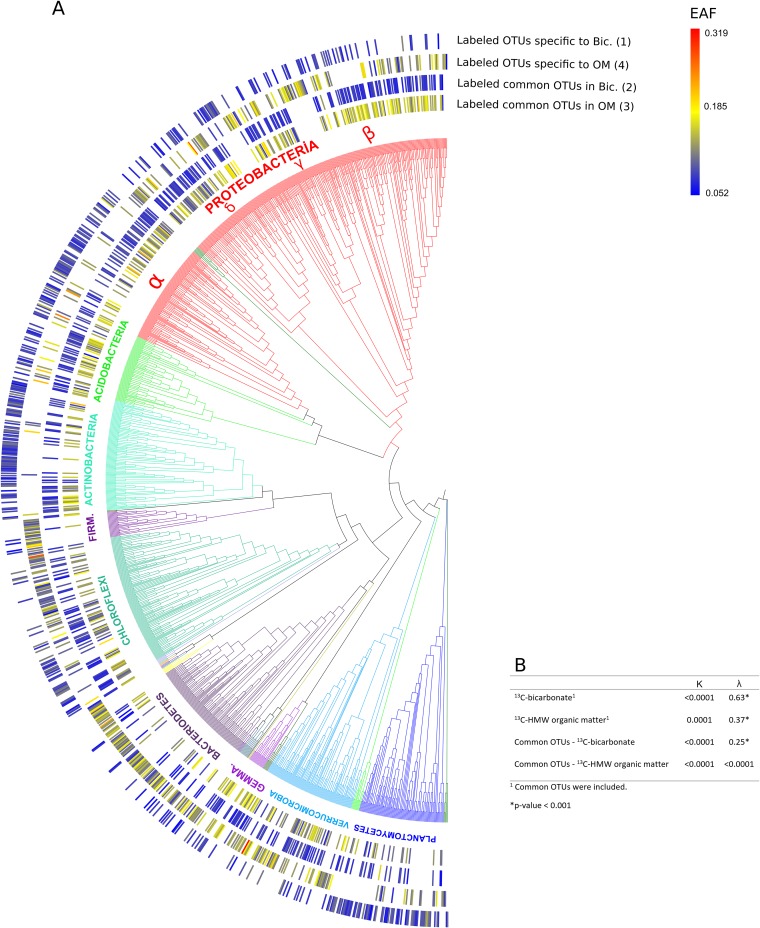
(A) (Left) Phylogeny of archaeal and bacterial taxa that assimilated the ^13^C-labeled substrate. The outer rings correspond to EAF values within the same four groups of either overlapping or nonoverlapping ^13^C-labeled OTUs with different treatments indicated in Fig. 4. (Right) Heat map of ^13^C isotope incorporation. (B) Table showing the results of phylogenetic signal tests (Blomberg's *K* and Pagel's λ) together with corresponding *P* tests. The outermost dark green branches on the lower left of the tree correspond to the few archaeal taxa that were ^13^C labeled (Table 1).

qSIP can also be used to reveal phylogenetic distribution patterns of functional traits involved in carbon substrate utilization (25). Our results show that all 798 labeled OTUs in the [^13^C]bicarbonate incubation (Fig. 4A) had significantly nonrandom phylogenetic distributions (λ = 0.63) (Fig. 5B), in accord with autotrophic traits exhibiting strong phylogenetic signals in a prior study (29). In contrast, all 823 labeled OTUs in the ^13^C-labeled HMW organic matter incubations (Fig. 4A) exhibited a significant but lower phylogenetic signal (λ = 0.37) (Fig. 5B). The weaker phylogenetic signal in heterotrophic populations could be explained by autotrophic and/or mixotrophic groups having more-conserved phylogeny than heterotrophic microbes, whose EAF values were randomly distributed throughout the phylogenetic tree (Fig. 5).

Microbial taxa affiliated with Verrucomicrobia were also well represented in the heterotrophic turnover of labeled organic matter (89 labeled OTUs [Table 1]). Several uncultivated OTUs affiliated with the Verrucomicrobia OPB35 soil clade were actively involved in HMW organic matter turnover derived from carbon fixation (EAF, 0.08 to 0.31). This is consistent with the traits of Verrucomicrobia, including their ability to degrade carbohydrates such as cellulose, mannan, and xylan in many environments (30, 31).

Actinobacteria OTUs specific to ^13^C-labeled bicarbonate incubations clustered into phylogenetically distinct clades (Fig. 5A), indicating carbon fixation in some lineages of aquatic Actinobacteria. This is consistent with another SIP study showing that Actinobacteria are responsible for carbon fixation in soil (32). Actinobacteria are also commonly observed to be involved in the degradation of plant- and alga-derived organic matter (33, 34). The uncultivated OTU affiliated with the MB-A2-108 clade in Actinobacteria had the highest EAF (0.11) within this phylum. Our results suggest that certain clades of Actinobacteria degrade the organic matter derived from chemolithoautotrophs, while other groups perform carbon fixation (see Fig. S5 in the supplemental material).

Microbial taxa affiliated with the Chloroflexi were also well represented in the heterotrophic turnover of labeled organic matter (108 OTUs [Table 1]). Chloroflexi are especially important for carbon cycling, ranging from anoxygenic phototrophy to organohalide respiration (35). They can degrade cellulose, xylose, starch, long-chain sugars, and pyrogallol, as well as utilizing oxidative phosphorylation and/or acetate fermentation for heterotrophic growth (35). Several DNA-SIP studies have also identified their important role in cellulose turnover in soil (31, 36). The freshwater pond sediments described in this study span a steep O_2_ gradient (37) sampled at the sediment-water interface that would have included suboxic or anoxic regions of sediment supporting the growth of anaerobic Chloroflexi. Indeed, uncultivated OTUs affiliated with the anaerobic Anaerolineaceae exhibited a relatively large amount of complex organic matter turnover and assimilation (EAF, 0.05 to 0.28).

There were a relatively high number of OTUs affiliated with Chloroflexi in the dark [^13^C]bicarbonate incubation (Table 1), suggesting the possibility that some of these OTUs may be capable of carbon fixation in the absence of sunlight. To our knowledge, no cultured Chloroflexi have been identified as chemolithoautotrophs. However, metagenomic data have shown that the RBG-2 and RBG-1351 groups, belonging to the GIF9 and GIF3 orders (formerly classes) of Chloroflexi, respectively, may be capable of chemoautotrophic growth via the Wood-Ljungdahl pathway (35). In addition, some members of the SAR202 clade in the dark ocean had low levels of DIC uptake, as determined by catalyzed reporter deposition–fluorescence *in situ* hybridization combined with microautoradiography (MICRO-CARD-FISH) (38). There are also studies reporting their utilization of labile and recalcitrant organic compounds (39). A study applying SIP of lipid biomarkers with DNA-based analysis of microbial communities to a shallow hydrothermal system showed that most of the fatty acids of Chloroflexi were labeled with [^13^C]bicarbonate, raising the possibility of chemolithoautotrophic growth (40). Interestingly, our qSIP results also showed that an OTU affiliated with the Chloroflexi GIF3 order had greater atomic enrichment (EAF, 0.12) than most of the OTUs in the [^13^C]bicarbonate incubation, supporting the possibility of chemolithoautotrophic growth.

We detected one labeled OTU of the uncultivated group Bathyarchaeota (EAF, 0.12) in the ^13^C-labeled HMW organic matter incubation. A phylogenetic cluster of Bathyarchaeota contains the genes for methane production determined from a genome-centric study (41); on the other hand, another study showed that they were able to degrade complex organic compounds and to produce acetate via a reductive acetyl coenzyme A (acetyl-CoA) pathway, suggesting that the Bathyarchaeota are a diverse phylum consisting of organotrophic-heterotrophic fermenters, methanogens, and autotrophic acetogens (42). Hence, our experimental qSIP data support genome-centric studies that indicate that Bathyarchaeota are anaerobic heterotrophs utilizing detrital organic matter and that they may be able to utilize HMW organic matter for growth.

Many of the low-abundance and uncultivated microbial populations detected in this study (e.g., Omnitrophica, Latescibacteria) exhibited heterotrophic utilization of HMW organic matter and were phylogenetically related to taxa from freshwater lakes and wetland ecosystems (see Fig. S7 in the supplemental material). Our results corroborate the proposed heterotrophic lifestyle deduced from the genes related to the degradation of amino acids and sugars that were detected in single-cell genomes of many of these rare uncultivated groups (13). Low-abundance taxa active in carbon cycling were rare relative to other groups, such as the Proteobacteria and Planctomycetes (Fig. 3). However, rare microbial taxa may represent a seed bank and may play an important ecological role when appropriate conditions arise (43, 44).

Our data are also consistent with a previous qSIP investigation showing the heterotrophic activity of the Latescibacteria in soil (14). For example, a genomic analysis of Latescibacteria (13) revealed their putative role in degrading organic matter in lake sediments (45), which supports our finding that they utilize high-molecular-weight organic substrates via heterotrophy in benthic freshwater settings. In the [^13^C]bicarbonate incubation, however, labeled OTUs were also affiliated with Latescibacteria (Table 1), suggesting that they may have been performing carbon fixation during the incubation. Genes that encode ribulose bisphosphate carboxylase, type III, have been found in the genome of Latescibacteria (13). These genomic data, together with our qSIP data, point toward possible mixotrophic growth for these groups. Interestingly, some OTUs affiliated with Latescibacteria and exhibiting potential mixotrophy were assigned to a specific clade (Fig. 5), suggesting that this phenotype might be restricted to certain lineages within this larger group.

### Assessing the biases of experimental conditions.

The *in situ* temperatures of the sediments (13.6 to 18.5°C in August and 1.4 to 8.3°C during the autumn months) were much lower than that for the room temperature incubation. The higher incubation temperature probably led to higher microbial activity than that in the *in situ* state, since warmer water temperatures facilitated increased mineralization of organic carbon in lake sediments (46). Moreover, the addition of bicarbonate to the samples likely altered the pH. However, the same amount of bicarbonate was added in the experiment and the control; thus, the pH should have remained constant for both, allowing us to identify OTUs that took up the [^13^C]bicarbonate (albeit at a pH different from that for *in situ* sediment). While the experimental conditions do not reflect the exact *in situ* state of the sediment, our data provide experimental evidence that under our chosen set of conditions, the activities of many microbial populations were reproducible and statistically significant.

### Conclusions.

Our results provide a direct link between carbon fixation by AOA and AOB and subsequent organic matter turnover by diverse uncultivated heterotrophic bacteria and archaea in a benthic lacustrine ecosystem. The dominance of chemolithoautotrophic AOA over AOB, as well as the incorporation of both ammonia oxidizers with ^13^C-labeled bicarbonate, revealed that AOA play a crucial role in dark primary production, fueling the benthic microbial loop under dark conditions. Several rare groups were involved in HMW organic matter turnover, indicating that they, too, play a role in benthic carbon cycling. Our data identified the turnover rates of the taxa responsible for dark primary production in freshwater sediments and demonstrated the ecological role of many taxa that were previously known solely on the basis of genomic signatures. Future qSIP time series studies may provide valuable insights into isotope turnover dynamics in benthic ecosystems.

## MATERIALS AND METHODS

### Stable isotope probing incubation.

Surface sediments (upper ∼2 cm) were collected from a freshwater pond (48°35′15″N, 12°4′38″E) near Landshut, Germany. Samples for extraction of ^13^C-enriched organic matter were obtained in August 2017, whereas samples for qSIP incubation were collected in October (for the [^13^C]bicarbonate incubation) and December (for the ^13^C-enriched high-molecular-weight [HMW] organic matter incubation) 2017. Substrates were added immediately after sample collection. The sediments exhibited dynamic seasonal changes in total inorganic carbon (TOC) levels, with higher values in the summer (average, 10.09% ± 0.36%) than in the winter (average, 8.5% ± 0.22%) (47). The carbon-to-nitrogen ratio (C/N) is nearly constant in space and time (January 2015, 13.2% ± 0.8%; August 2016, 13.8% ± 1.3%; 1σ uncertainties) (47). The bottom water temperatures ranged from 13.6 to 18.5°C, 4.6 to 8.3°C, and 1.4 to 3.3°C in August, October, and December, respectively (47).

Surface sediment samples collected for organic matter extraction in August 2017 were incubated in the dark in crimp-sealed glass vials (38 g with 4 ml of headspace) for 2.5 months at room temperature and were amended with 100 mM unlabeled and 99% ^13^C-labeled sodium bicarbonate (NaHCO_3_; Sigma-Aldrich, St. Louis, MO, USA). All sediments were transferred to 50-ml Lysing Matrix E tubes (MP Biomedicals, Solon, OH, USA) containing 1.4-mm ceramic spheres, 0.1-mm silica spheres, and one 4-mm glass sphere and were then homogenized for 40 s in a Fast-Prep 5G homogenizer (MP Biomedicals, Solon, OH, USA) at a speed of 6 m/s in the presence of 10 ml lysing buffer containing (for a 50-ml solution) 4 ml of C1 lysing buffer (MoBio, Carlsbad, CA), 0.8 ml 10% SDS, 7.2 ml 100% ethanol, and 38 ml 1 M disodium hydrogen phosphate (Na_2_HPO_4_). Afterwards, samples were heated for 2 min at 99°C and were then frozen twice (two freeze-thaw cycles). Samples were centrifuged for 10 min at 4,200 × *g*, and the supernatant was then transferred to Amicon filters (molecular weight cutoff [MWCO], 50 kDa; Millipore, St. Louis, MO, USA) to concentrate the HMW organic matter. Concentrated organic matter was kept at −20°C until it was added back to the sediments for the qSIP experiments.

Surface sediments from the pond were amended with ^13^C-labeled HMW organic matter and 10 mM ^13^C-labeled bicarbonate and were incubated for 1 week in the dark (Fig. 1). Control incubations in which samples were amended with an unlabeled substrate were also carried out at a substrate concentration equivalent to that for the experimental incubations. Each incubation was performed in 20-ml crimp-sealed glass vials (20 g of sediment) or paraffin-sealed petri dishes (80 g of sediment; 100 by 15 mm), with 10 mm and 20 mm headspace, respectively.

DNA from the samples was extracted using an established protocol (48) with minor modifications. In brief, 0.5 g of the sediment was transferred to 2-ml Lysing Matrix E tubes containing 1.4-mm ceramic spheres, 0.1-mm silica spheres, and one 4-mm glass sphere (MP Biomedicals, Solon, OH, USA) following each incubation. One milliliter of the lysing buffer (see above) was added and homogenized for 40 s in a Fast-Prep 5G homogenizer at a speed of 6 m/s. Then the supernatant containing the DNA was purified with a MoBio DNA extraction kit. Extracted DNA was quantified by using the Qubit double-stranded DNA (dsDNA) high-sensitivity assay kit and a Qubit 3.0 fluorometer (Invitrogen, Eugene, OR, USA).

### Density gradient centrifugation and gradient fraction.

DNA samples were prepared for density gradient centrifugation according to a previously defined protocol for DNA-qSIP (49, 50) with minor modifications. Density gradient centrifugations were carried out in a TLN-100 Optima MAX-TL ultracentrifuge (Beckman Coulter, Brea, CA, USA) with a near-vertical rotor at 18°C for 72 h at 165,000 × *g*. Fifty microliters of DNA, ranging from 0.5 μg to 1.5 μg, which was within the range of proposed values (50), was added to a solution of cesium chloride (CsCl) and gradient buffer (0.1 M Tris, 0.1 M KCl, and 1 mM EDTA) in order to achieve a starting density of 1.70 g ml^−1^ in 3.3-ml OptiSeal polyallomer tubes (Beckman Coulter, Brea, CA, USA). After ultracentrifugation, the density gradients were fractionated into 15 equal fractions of 200 μl from the bottoms of OptiSeal polyallomer tubes by using a syringe pump and fraction recovery system (Beckman Coulter, Brea, CA, USA). The densities of these fractions were measured with an AR200 digital refractometer (Reichert Analytical Instruments, Depew, NY, USA). DNA was precipitated from the fractions overnight at room temperature using 2 volumes of polyethylene glycol with 2 μl (10 mg ml^−1^) glycogen. DNA was pelleted by centrifugation (13,000 × *g*, 40 min), washed with 70% ethanol, and resuspended with 30 μl molecular-grade (diethyl pyrocarbonate [DEPC]-treated) water. DNA was quantified fluorometrically using a Qubit fluorometer.

### qPCR and high-throughput 16S rRNA gene sequencing.

Universal primers 515F and 806R, targeting the V4 hypervariable region of 16S rRNA genes (51), were used in quantitative PCR (qPCR) to determine shifts in the peak buoyant density (BD) of DNA for each incubation. qPCRs were carried out in 20-μl solutions containing 10.4 μl SsoAdvanced SYBR green PCR buffer (Bio-Rad, Hercules, CA, USA), 0.4 μl of 10 mM primer, 6.8 μl of nuclease-free water, and 2 μl of the DNA template. All reactions were performed with a two-step protocol in a CFX Connect real-time PCR system (Bio-Rad, Hercules, CA, USA), including an enzyme activation step at 95°C for 3 min, followed by 40 cycles of denaturation at 95°C for 15 s and then annealing at 55°C for 30 s. Each density fraction was also screened using qPCR for ammonia-oxidizing archaea and bacteria with primer pairs targeting the ammonia monooxygenase subunit A (*amoA*) gene according to previously published assays (52, 53). Briefly, all reactions for archaeal *amoA* genes were performed with a three-step protocol including an enzyme activation step at 95°C for 3 min, followed by 40 cycles of denaturation at 95°C for 30 s, annealing at 53°C for 1 min, and extension at 72°C for 1 min using a CFX Connect real-time PCR system. The PCR conditions for bacterial *amoA* genes were the same except that the annealing temperature was 60°C. As in prior studies (54, 55), qPCR standards consisted of 10-fold dilution series of the genes of interest that were PCR amplified from the sample for 40 cycles using the same primers. Prior to the creation of the dilution series, the amplified standard was gel extracted and quantified with a Qubit instrument. The reaction efficiencies in all qPCR assays were between 90% and 110%, with an *r*^2^ of >98 for the standards.

Two PCR amplicons from each density fraction (technical replicates to reduce PCR bias) were pooled and subjected to dual-indexed barcoded sequencing of 16S rRNA gene amplicons on the Illumina MiniSeq system (56). High-throughput sequencing of the barcoded 16S amplicons was carried out using an Illumina (San Diego, CA, USA) MiniSeq system with a Mid-Output kit (two 150-bp paired-end reads) at the Geo-Bio Center of Ludwig-Maximilians Universität München using a dual-index custom primer protocol optimized for the MiniSeq platform (56). In order to account for the influence of contamination, we included barcoded 16S amplicons in the sequencing run to detect potential sources of contamination, such as aerosols (laboratory dust) and kit reagents (DNA extraction blanks).

### Bioinformatic analysis.

The MiniSeq reads were quality trimmed and assembled using USEARCH, version 10.0.240, with the default parameters (57), resulting in 6.8 million quality-checked V4 reads. Reads were then *de novo* clustered at 97% identity using UPARSE; OTUs represented by a single sequence were discarded (58). Taxonomic assignments were generated by QIIME, version 1.9.1 (59), using the implemented BLAST method against the SILVA rRNA gene database, release 128 (60). After that, only OTUs more abundant than 12 sequences in total in each replicate for the control and SIP-labeled fractions were selected for further study (54). In total, 1,271 and 945 OTUs from samples amended with bicarbonate and HMW organic matter, respectively, remained. OTUs detected in the contaminant data sets (e.g., laboratory dust, extraction blanks) were removed from all downstream analyses if the total number of contaminants in each OTU was greater than the total number of corresponding OTUs. With this “cleaned” data set, 1,231 OTUs from the samples with bicarbonate added and 931 OTUs from those with HMW organic matter added were used for downstream analyses. The same bioinformatic workflow consistently recovered accurate 16S rRNA gene OTU richness from mock communities sequenced on the MiniSeq system (56). We are thus confident that the OTUs in this study, produced using the workflow described above, represent coherent taxonomic units and are not significantly influenced by sequencing or clustering errors.

The observed excess atom ^13^C enrichment fraction (EAF) was calculated for each taxon according to a previously described study (14) using a qSIP workflow embedded in the HTS-SIP R package (61). Weighted average densities were calculated for each taxon's DNA in the control incubation (^12^C added) and in the experimental incubation (^13^C added) as described by Hungate et al. (14) to estimate the excess atom fraction of ^13^C for each OTU. To calculate the bootstrap confidence intervals (CI) for significant isotopic incorporation, bootstrap replicates (*n* = 1,000) were run with the HTS-SIP R package (61); an OTU was considered to be ^13^C labeled if the 90% CI was above the 0% EAF cutoff (14).

For phylogenetic analyses, OTUs of interest and their closest BLAST hits were selected to construct phylogenetic trees in SeaView (62) following alignment with MUSCLE (63). Maximum likelihood (ML) with a general time-reversible (GTR) substitution model was performed with PhyML, version 3.0 (64). Trees were visualized and edited using iTOL (65). Statistical analyses and plots were performed using RStudio, version 3.3.0 (66), with the vegan package (67). Blomberg's *K* (68) and Pagel's λ (69) tests for significantly nonrandom phylogenetic distributions of carbon utilization from qSIP were calculated using the R package phylosignal (70). Both indices test species' traits under a Brownian motion model (BM) of trait evolution; that is, they test whether the distribution of traits across different phylogenetic groups is random or nonrandom. The BM assigns a value of 0 to indicate phylogenetic independence (random phylogenetic distribution of traits) and values close to 1 for a strong phylogenetic signal (nonrandom phylogenetic distribution of traits). These tests were used in previous qSIP studies to assign putative ecological functions to specific phylogenetic clades (25, 71, 72).

### Accession number(s).

Sequence data were entered into the NCBI Sequence Read Archive under BioProject ID PRJNA418911.

## Supplementary Material

Supplemental file 1
