# Supplementary material for: Linking Uncultivated Microbial Populations and Benthic Carbon Turnover by Using Quantitative Stable Isotope Probing
Source: Appl Environ Microbiol. 2018 Aug 31;84(18):e01083-18. doi: 10.1128/AEM.01083-18 (PMC6122004; doi:10.1128/AEM.01083-18)
Supplement: Supplemental file 1 [file zam018188719s1.pdf]

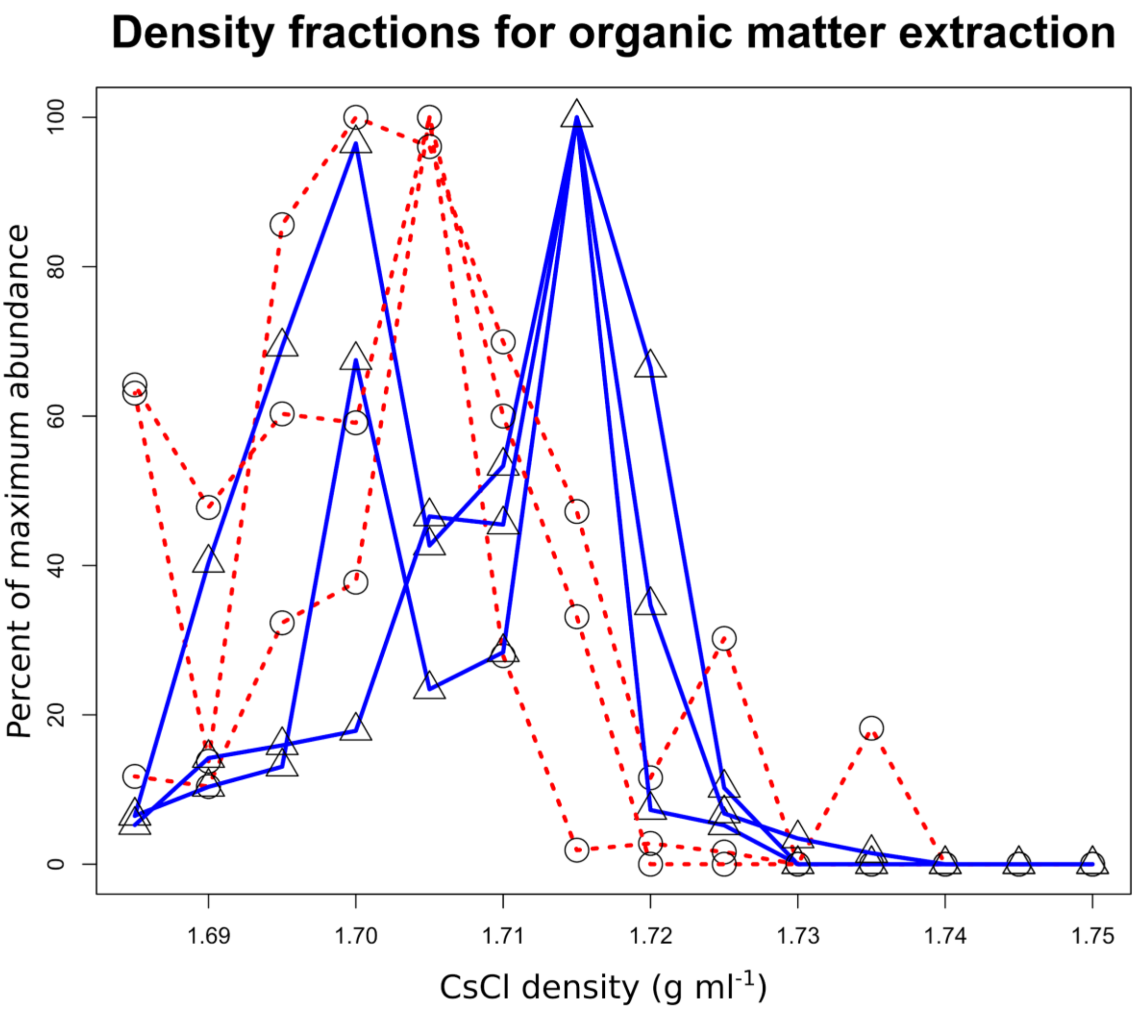

Supplementary Fig. 1. Plots representing the shifts in the BD of total DNA in each density fractions in glass vials incubated with ^{13}C-labeled bicarbonate for 2.5-month period. Red colored dashed lines with circles represent the control (^{12}C-labeled fractions) and blue colored continuous lines with triangles represent the experiment (^{13}C-labeled fractions). The x axis shows the CsCl density of all fractions and the y axis represents the relative abundances calculated from their maximal abundance. The two separate peaks in the ^{13}C-labeled incubation likely derive from (1) unlabeled DNA from plants and detrital extracellular DNA (unlabeled peak to at ca. 1.70 g mL^{-1}) and (2) labeled DNA from actively growing microbes and their consumers (labeled peak at ca. 1.72 g mL^{-1}).

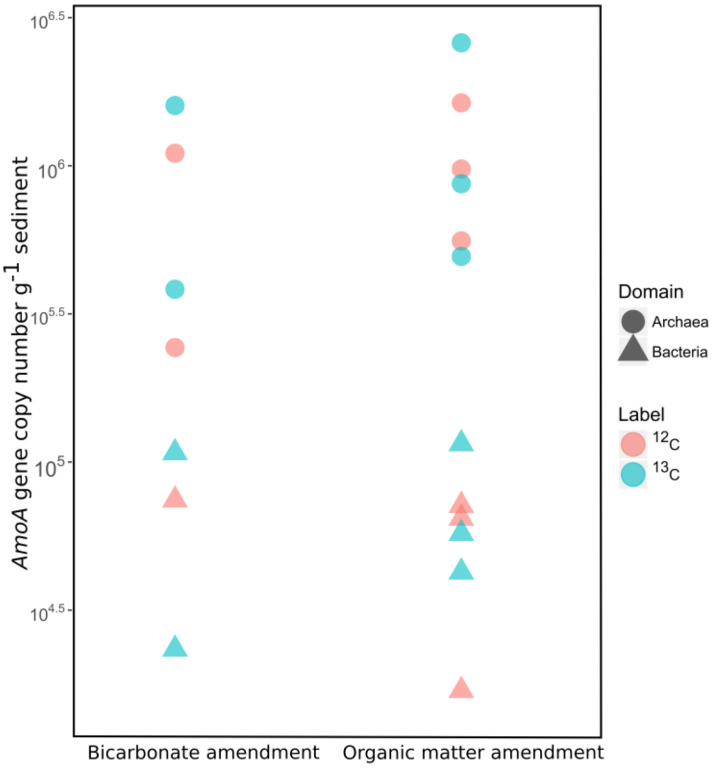

Supplementary Fig. 2. Archaeal and bacterial *amoA* gene copy numbers per gram sediment in labeled and unlabeled incubation settings. Note that archaeal *amoA* genes were more abundant than bacterial *amoA* genes in freshwater sediment with the average AOA:AOB ratio of ~15 in the incubation amended with ^{13}C-labeled bicarbonate and ~19 in the incubation amended with ^{13}C-enriched organic matter for 1 week period.

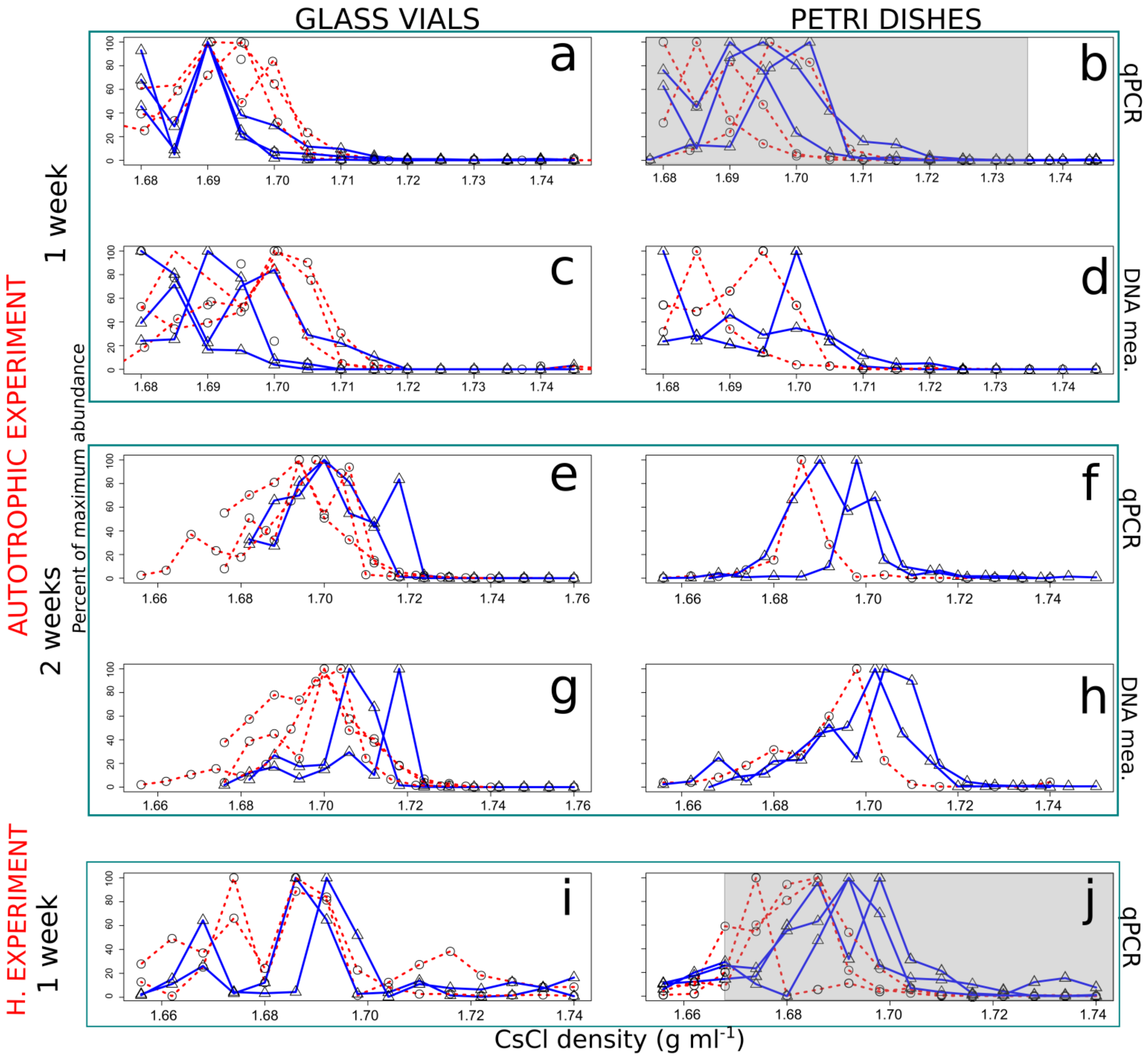

Supplementary Fig. 3. Plots representing the shifts in the BD of density fractions in glass vials and petri dishes. Red colored dashed lines with circles represent the control (^{12}C-labeled fractions) and Blue colored continuous lines with triangles represent the experiment (^{13}C-labeled fractions). The x axis shows the CsCl density of all fractions and the y axis represents the relative abundances calculated from their maximal abundance. Gray shading and symbols filled with black color indicate the fractions used for sequencing. Abbreviations: BD, buoyant density, DNA mea., lines plotted based on DNA measurements, qPCR, lines plotted based on quantitative PCR, H., Heterotrophic.

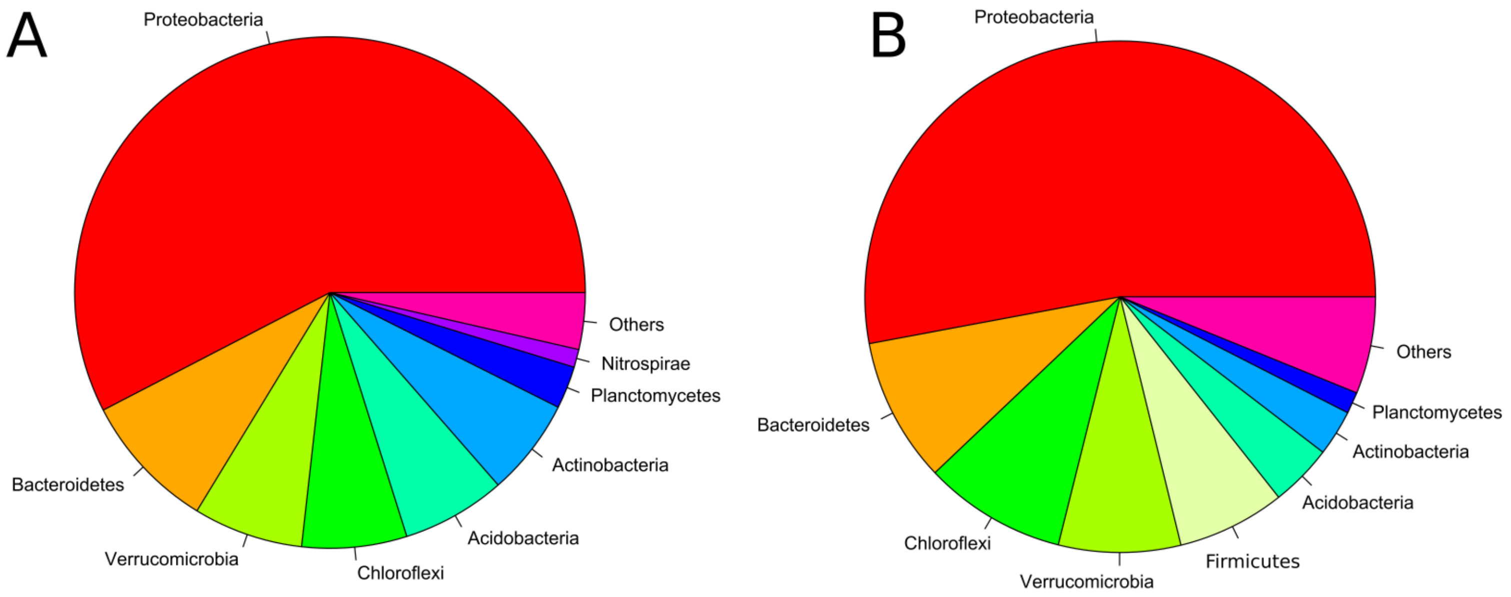

Supplementary Fig. 4. Pie charts representing the total bacterial community of the density fractions regardless of assimilation of the ^{13}C-labeled bicarbonate (A) and HMW organic matter (B).

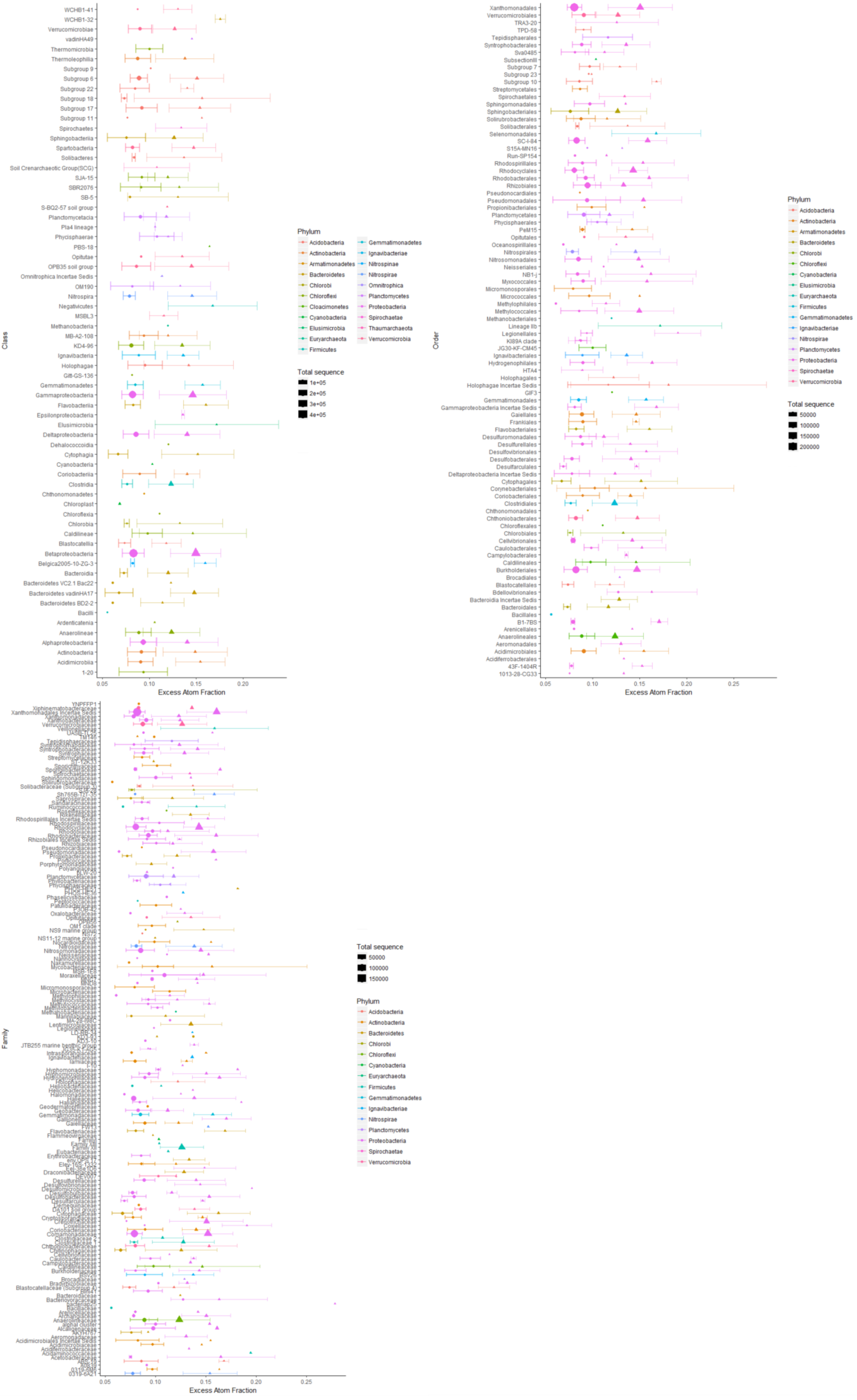

WCHB1-41
WCHB1-32
Verrucomicrobiae
vadinHA49
Thermomicrobia
Thermoleophilia
Subgroup 9
Subgroup 6
Subgroup 22
Subgroup 18
Subgroup 17
Subgroup 11
Spirochaetes
Sphingobacteriia
Spartobacteria
Solibacteres
Soil Crenarchaeotic Group(SCG)
SJA-15
SBR2076
SB-5
S-BQ2-57 soil group
Planctomycetacia
Pla4 lineage
Phycisphaerae
PBS-18
Opitutae
OPB35 soil group
Omnitrophica Incertae Sedis
OM190
Nitrospira
Negativicutes
MSBL3
Methanobacteria
MB-A2-108
KD4-96
Ignavibacteria
Holophagae
Gitt-GS-136
Gemmatimonadetes
Gammaproteobacteria
Flavobacteriia
Epsilonproteobacteria
Elusimicrobia
Deltaproteobacteria
Dehalococcoidia
Cytophagia
Cyanobacteria
Coriobacteriia
Clostridia
Chthonomonadetes
Chloroplast
Chloroflexia
Chlorobia
Caldilineae
Blastocatellia
Betaproteobacteria
Belgica2005-10-ZG-3
Bacteroidia
Bacteroidetes VC2.1 Bac22
Bacteroidetes vadinHA17
Bacteroidetes BD2-2
Bacilli
Ardenticatenia
Anaerolineae
Alphaproteobacteria
Actinobacteria
Acidimicrobiia
1-20
Class
0.05
0.10
0.15
0.20
Excess Atom Fraction
Phylum
Acidobacteria
Actinobacteria
Armatimonadetes
Bacteroidetes
Chlorobi
Chloroflexi
Cloacimonetes
Cyanobacteria
Elusimicrobia
Euryarchaeota
Firmicutes
Gemmatimonadetes
Ignavibacteriae
Nitrospinae
Nitrospirae
Omnitrophica
Planctomycetes
Proteobacteria
Spirochaetae
Thaumarchaeota
Verrucomicrobia
Total sequence
1e+05
2e+05
3e+05
4e+05
Xanthomonadales
Verrucomicrobiales
TRA3-20
TPD-58
Tepidisphaerales
Syntrophobacterales
Sva0485
SubsectionIII
Subgroup 7
Subgroup 23
Subgroup 10
Streptomycetales
Spirochaetales
Sphingomonadales
Sphingobacteriales
Solirubrobacterales
Solibacterales
Selenomonadales
SC-I-84
S15A-MN16
Run-SP154
Rhodospirillales
Rhodocyclales
Rhodobacterales
Rhizobiales
Pseudonocardiales
Pseudomonadales
Propionibacteriales
Planctomycetales
Phycisphaerales
PeM15
Opitutales
Oceanospirillales
Nitrospirales
Nitrosomonadales
Neisseriales
NB1-j
Myxococcales
Micromonosporales
Micrococcales
Methylophilales
Methylococcales
Methanobacteriales
Lineage IIb
Legionellales
KI89A clade
JG30-KF-CM45
Ignavibacteriales
Hydrogenophilales
HTA4
Holophagales
Holophagae Incertae Sedis
GIF3
Gemmatimonadales
Gammaproteobacteria Incertae Sedis
Gaiellales
Frankiales
Flavobacteriales
Desulfuromonadales
Desulfurellales
Desulfovibrionales
Desulfobacterales
Desulfarculales
Deltaproteobacteria Incertae Sedis
Cytophagales
Corynebacteriales
Coriobacteriales
Clostridiales
Chthonomonadales
Chthoniobacterales
Chloroflexales
Chlorobiales
Cellvibrionales
Caulobacterales
Campylobacterales
Caldilineales
Burkholderiales
Brocadiales
Blastocatellales
Bdellovibrionales
Bacteroidia Incertae Sedis
Bacteroidales
Bacillales
B1-7BS
Arenicellales
Anaerolineales
Aeromonadales
Acidimicrobiales
Acidiferrobacterales
43F-1404R
1013-28-CG33
Order
0.05
0.10
0.15
0.20
0.25
Excess Atom Fraction
Phylum
Acidobacteria
Actinobacteria
Armatimonadetes
Bacteroidetes
Chlorobi
Chloroflexi
Cyanobacteria
Elusimicrobia
Euryarchaeota
Firmicutes
Gemmatimonadetes
Ignavibacteriae
Nitrospirae
Planctomycetes
Proteobacteria
Spirochaetae
Verrucomicrobia
Total sequence
50000
100000
150000
200000
Family
0.05
0.10
0.15
0.20
0.25
Excess Atom Fraction
Total sequence
50000
100000
150000
Phylum
Acidobacteria
Actinobacteria
Bacteroidetes
Chlorobi
Chloroflexi
Cyanobacteria
Euryarchaeota
Firmicutes
Gemmatimonadetes
Ignavibacteriae
Nitrospirae
Planctomycetes
Proteobacteria
Spirochaetae
Verrucomicrobia

Supplementary Fig. 5 Mean ± standard error ^{13}C EAF in the DNA of grouped taxa within class (Left top) order (Right top) and family (Bottom left) level after incubation with ^{13}C-labeled HMW organic matter (triangle) and ^{13}C-labeled (circle). Each taxonomic level was colored by phylum and the relative abundance of each taxonomic level is proportional to the size of symbols.

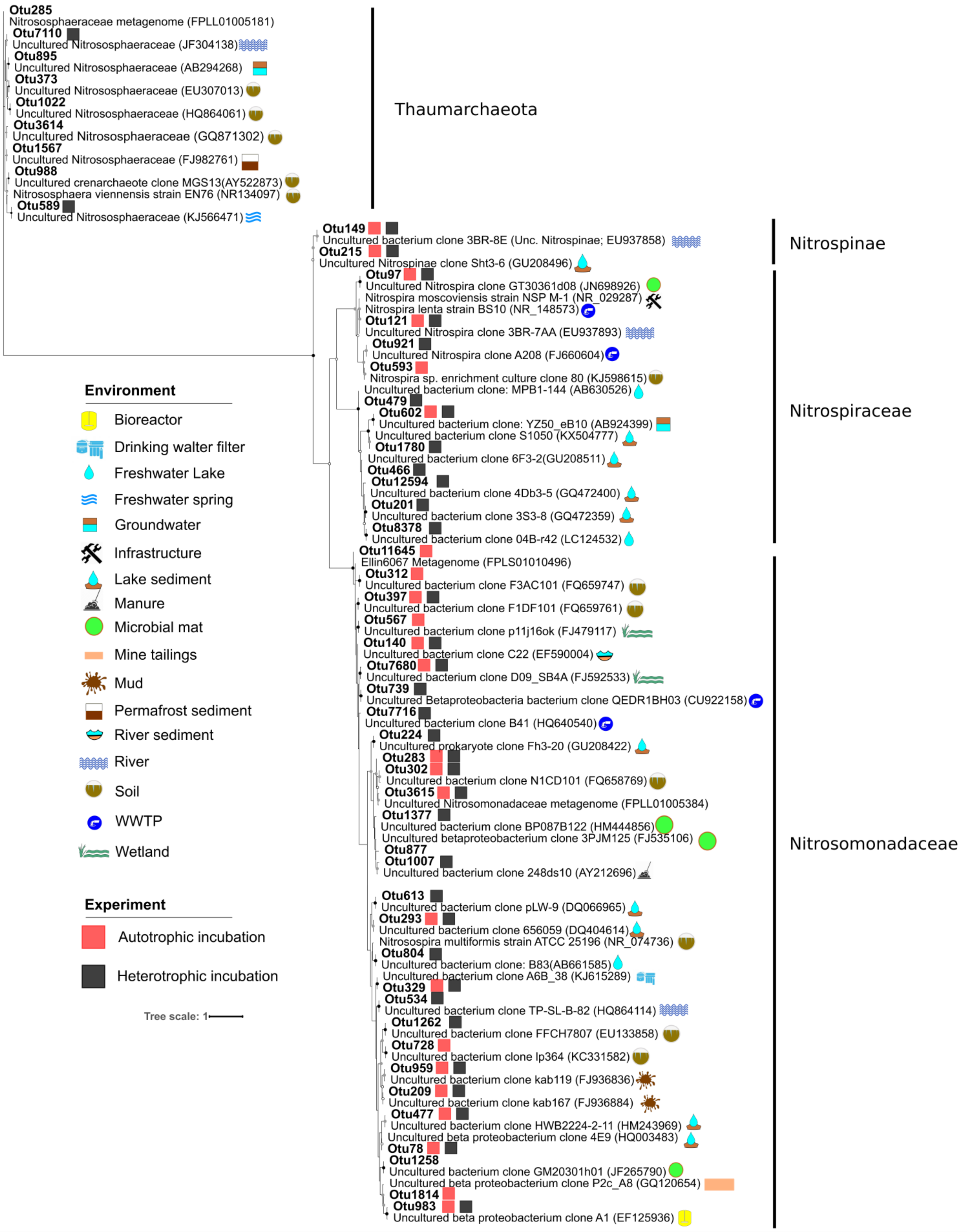

Supplementary Fig. 6. Maximum likelihood phylogenies (PhyML) of betaproteobacterial ammonia-oxidizers and nitrite-oxidizing *Nitrospira* and *Nitrospinae* 16S rRNA genes sharing 97% sequence identity with OTUs after a one-week incubation amended with ^{13}C-labeled bicarbonate. OTUs which assimilated the labeled substrate represented as red stars next to each OTU. Black circles at nodes represent bootstrap support of 90%, gray circles represent bootstrap support from 70% to 90%, and white circles represent bootstrap support from 70% to 50%. Numbers represent NCBI GI accession IDs.

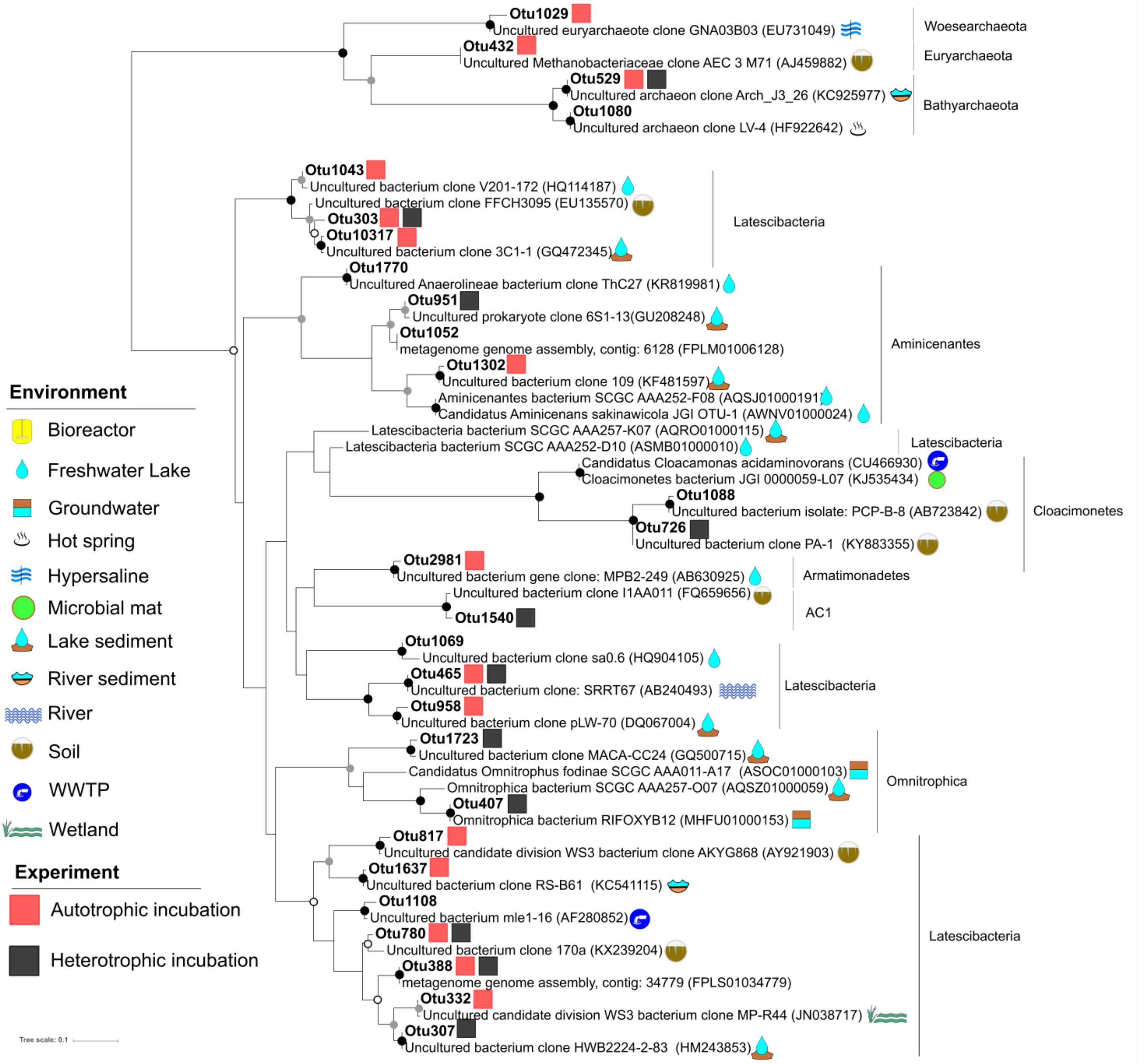

Supplementary Fig. 7. Maximum likelihood phylogenies (PhyML) of MDM 16S rRNA genes sharing 97% sequence identity with OTUs after a one-week incubation amended with ^{13}C-enriched organic matter. OTUs which assimilated the labeled organic matter and bicarbonate represented as red stars and black squares next to each OTU, respectively. Black circles at nodes represent bootstrap support of 90%, gray circles represent bootstrap support from 70% to 90%, and white circles represent bootstrap support from 70% to 50%. Numbers represent NCBI GI accession IDs.

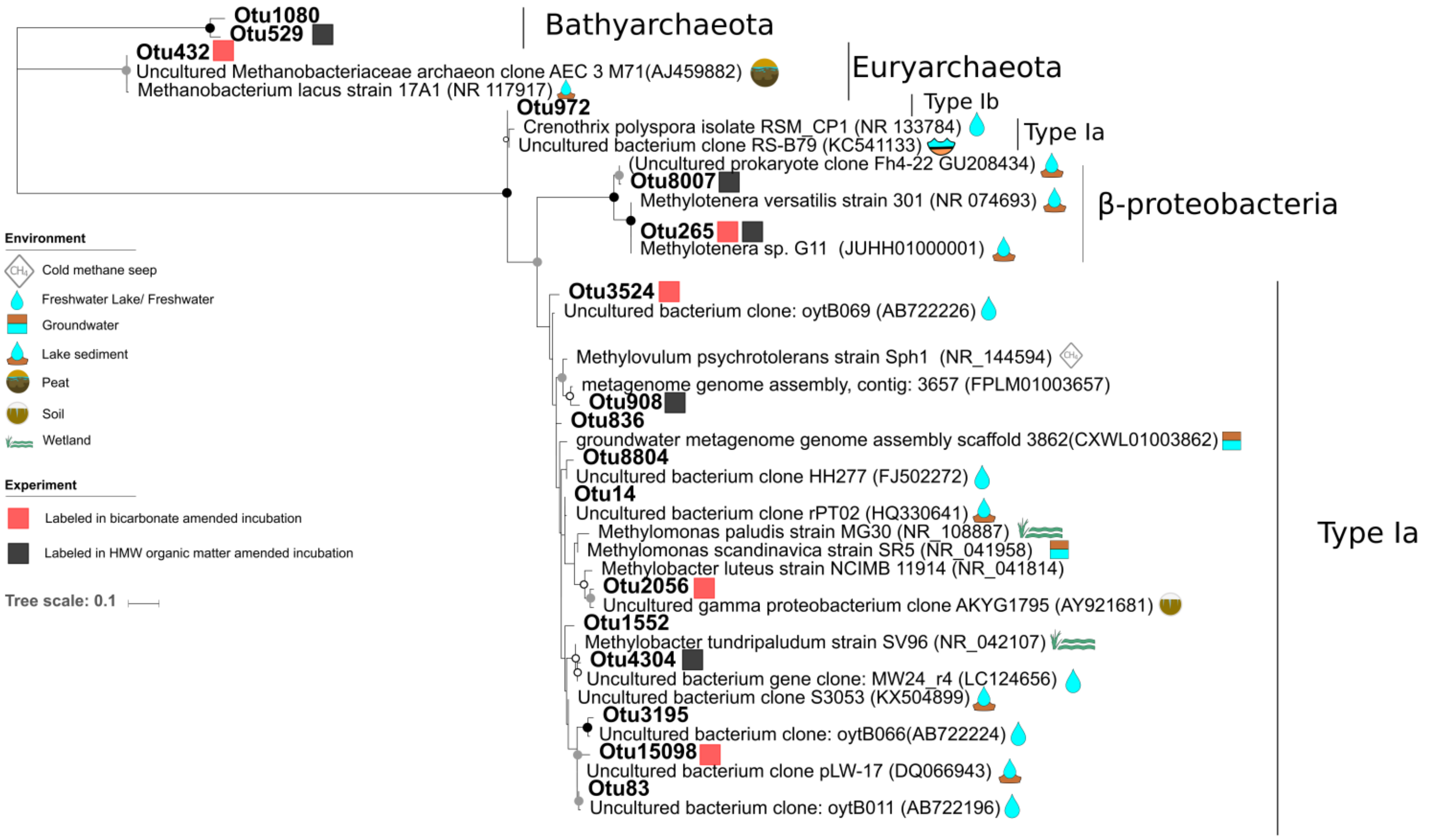

Supplementary Fig. 8. Maximum likelihood phylogenies (PhyML) of methylotrophs and methanogens 16S rRNA genes sharing 97% sequence identity with OTUs after a one-week incubation amended with ^{13}C-labeled HMW organic matter. OTUs which assimilated the labeled substrate represented as red stars next to each OTU. Black circles at nodes represent bootstrap support of 90%, gray circles represent bootstrap support from 70% to 90%, and white circles represent bootstrap support from 70% to 50%. Numbers represent NCBI GI accession IDs.
